# Study of Copper-Nickel Nanoparticle Resistive Ink Compatible with Printed Copper Films for Power Electronics Applications

**DOI:** 10.3390/ma14227039

**Published:** 2021-11-20

**Authors:** Jiri Hlina, Jan Reboun, Ales Hamacek

**Affiliations:** Department of Materials and Technology, Faculty of Electrical Engineering, University of West Bohemia, Univerzitni 8, 301 00 Pilsen, Czech Republic; jreboun@fel.zcu.cz (J.R.); hamacek@fel.zcu.cz (A.H.)

**Keywords:** copper, nickel, electrical properties, thick-film resistor, contact resistance, resistive ink

## Abstract

This paper is focused on copper–nickel nanoparticle resistive inks compatible with thick printed copper (TPC) technology, which can be used for power substrate manufacturing instead of conventional metallization techniques. Two types of copper–nickel inks were prepared and deposited by Aerosol Jet technology. The first type of ink was based on copper and nickel nanoparticles with a ratio of 75:25, and the second type of ink consisted of copper–nickel alloy nanoparticles with a ratio of 55:45. The characterization of electrical parameters, microstructure, thermal analysis of prepared inks and study of the influence of copper–nickel content on electrical parameters are described in this paper. It was verified that ink with a copper–nickel ratio of 55:45 (based on constantan nanoparticles) is more appropriate for the production of resistors due to low sheet resistance ~1 Ω/square and low temperature coefficient of resistance ±100·10^−6^ K^−1^ values. Copper–nickel inks can be fired in a protective nitrogen atmosphere, which ensures compatibility with copper films. The compatibility of copper–nickel and copper films enables the production of integrated resistors directly on ceramics substrates of power electronics modules made by TPC technology.

## 1. Introduction

Resistors represent one of the most used passive electronic components in power electronics. Typical applications of resistors in power electronics are shunt resistors or current sensing resistors [1]. The main requirements for these resistors are a low resistance, high stability over a wide range of temperatures and low temperature coefficient of resistance (TCR) [2]. These resistors are usually used in the discrete form such as wire-wound resistors or thick-film resistors soldered to the copper metallization of substrates because conventional techniques for power substrate manufacturing such as direct bonded copper (DBC)—based on the bonding of copper foil to ceramics through Cu-Cu_2_O eutectic [3,4]—or active metal brazing (AMB)—based on the connection of copper foil to ceramics with brazing alloy [5]—do not allow for a direct integration of passive components. Direct integration, in this case, means the printing of resistive material directly on substrates with copper metallization. It can improve the reliability of power substrates and increase heat transmission from the resistor to the substrate [6].

The direct integration of passive components is well established in conventional thick-film technology based on silver paste metallization used in a wide range of electronic applications. Silver pastes are mainly determined for firing in an oxidative atmosphere, which is usually common air [7]. However, silver metallization cannot meet power electronic substrates’ requirements because it is complicated and expensive to ensure the proper thickness of conductive traces for high current passing.

This issue can be solved by thick printed copper (TPC) technology, which also enables the direct integration of resistors and can be used for the manufacturing of power electronic substrates instead of conventional methods such as DBC or AMB [8,9]. Special copper pastes are used for TPC substrate production [10]. These pastes are sequentially screen-printed usually on alumina substrates and fired in a protective nitrogen atmosphere at temperatures higher than 900 °C [11,12]. In addition to the direct integration of resistors, TPC technology also has other benefits compared to the above-mentioned conventional technologies such as the high resolution of copper patterns, high endurance to thermal cycles, the formation of multilayer circuits including copper-filled vias and also the possibility of metallization on aluminum nitride (AlN) substrates for high-power applications [13,14]. The capabilities mentioned above predetermine TPC technology for the production of complex power electronics modules.

The necessity of copper film firing in a protective nitrogen atmosphere significantly complicates the direct integration of resistors on TPC substrates because standard resistive thick-film pastes are usually based on ruthenium compounds. These pastes are determined for firing in an oxidative atmosphere [15,16]. Currently, there are no commercially available thick-film resistive pastes compatible with printed copper films. Previous research has verified that some conventional thick-film resistive pastes can also be used for the formation of resistors on TPC substrates while using a combination of firing in an oxidative and protective nitrogen atmosphere [8]. However, this method brings the problems of high contact resistance between copper and resistive films, which results in a very difficult production of low-ohm resistors required for shunt or current sensing applications.

This problem can be solved using the resistive film based on copper–nickel alloy resistive ink, which enables firing in a nitrogen atmosphere. Using copper–nickel resistive film eliminates the necessity of a combination of firing in an oxidative and nitrogen atmosphere. There is a presumption of low contact resistance between copper and copper–nickel films due to the fact that both films are metal-based. The copper–nickel resistive film also shows low resistance and TCR [17,18]. Copper–nickel ink and the combination of the copper–nickel resistive film with printed copper have not been described in detail in the literature yet. Therefore, this paper is focused on the study of printed resistors based on the copper–nickel resistive film for power electronic applications.

## 2. Materials and Methods

Two copper–nickel inks with different ratios of copper and nickel were prepared for experiments in cooperation with the company Applied Nanotech (Austin, TX, USA). The first ink was prepared from 20–100 nm copper and 20–100 nm nickel particles which were mixed in a ratio of 75:25 (specimen CuNi 75:25). The second ink was prepared from 20–40 nm constantan particles (specimen CuNi 55:45). Constantan is a copper–nickel alloy with a ratio of 55:45. Copper, nickel and constantan nanoparticles were prepared by milling from bulk materials. Nanoparticles were diluted in an organic-based solvent based on a combination of 50 wt.% isopropyl alcohol, 5 wt.% 2-benzyloxyethanol and 15 wt.% 2-ethoxyethanol. The organic-based solvent formed 70 wt.% of the final inks. Combining solvents with low boiling points and high boiling points is appropriate for the Aerosol Jet deposition technique. The solvent with a low boiling point facilitates the formation of aerosol in an atomizer. In contrast, a solvent with a high boiling point reduces aerosol drying in the course of its carrying by sheath gas flow during deposition [19]. The solid content of nanoparticles in inks is 30 wt.%, which was verified by thermogravimetric analysis (TA Instruments SDT Q600). Copper–nickel ratios were confirmed by EDS analysis. The thermal properties of prepared inks were verified using differential scanning calorimetry (DSC).

**Figure 1:**
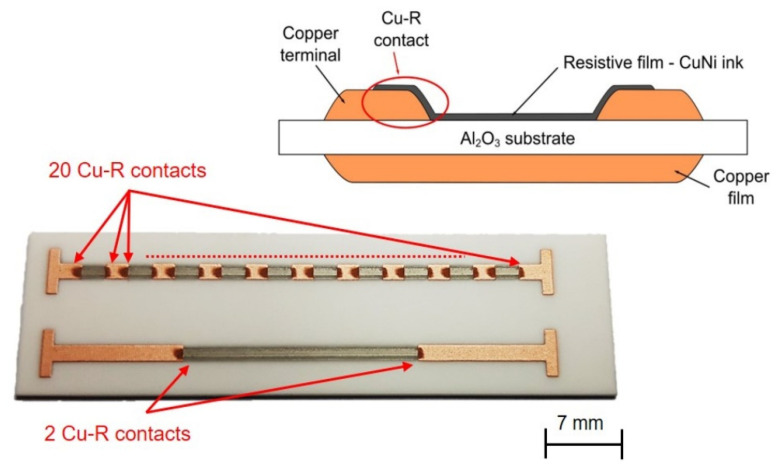
Formed IRP and the structure of the resistor with the copper–nickel resistive film. Cu-R contact means the contact between the copper film and copper–nickel resistive film, this contact causes contact resistance.

The behavior of copper–nickel resistive film in combination with TPC terminals was verified using an intermittent resistor pattern (IRP). This pattern is described in detail in [8]. IRP includes two patterns (Figure 1). The first pattern consists of ten two-square resistive structures and twenty contacts between resistive and copper film terminals. The second pattern contains one twenty-square resistive structure and two contacts between resistive and copper film terminals. The main purpose of IRP is also verifying the contact resistance between copper–nickel resistive films and copper terminals.

Copper–nickel resistive films were deposited using an Optomec Aerosol Jet 300-UP (Albuquerque, NM, USA) [20] and fired in a nitrogen atmosphere at a temperature of 960 °C with a 10 min dwell time on the peak temperature with the same firing profile as for copper films. The advantage of this is the full compatibility of copper–nickel and copper films, effortless manufacturing and co-firing capability. The parameters of Aerosol Jet deposition were as follows: wide nozzle head, 0.75 mm nozzle diameter, pneumatic atomizer, deposition head speed—4 mm·s^−1^, substrate heating—80 °C, nitrogen flows—atomizer—1200 sccm, exhaust—1000 sccm, and sheath gas—80 sccm. Experimental fully printed coper-nickel resistor specimens were produced using a standard thick-film resistor production process based on the printing and firing of terminals in the first step and printing and firing of the resistive film in the second step. The structure of the resistor with the copper–nickel resistive film is shown in Figure 1.

The temperature characteristics of produced specimens were measured in the range from 0 °C to 100 °C in a silicon oil calibration thermostatic bath Lauda PJL 12 using the four-wire resistance measurement method. The TCR of formed specimens was calculated according to the following formula:

TCR = (1/R_ref_) × (R − R_ref_)/(T − T_ref_) (K^−1^) (1)

where R is the resistance value, R_ref_ is the resistance value at 0 °C and T_ref_ is the reference temperature (0 °C).

## 3. Results and Discussion

**Figure 2:**
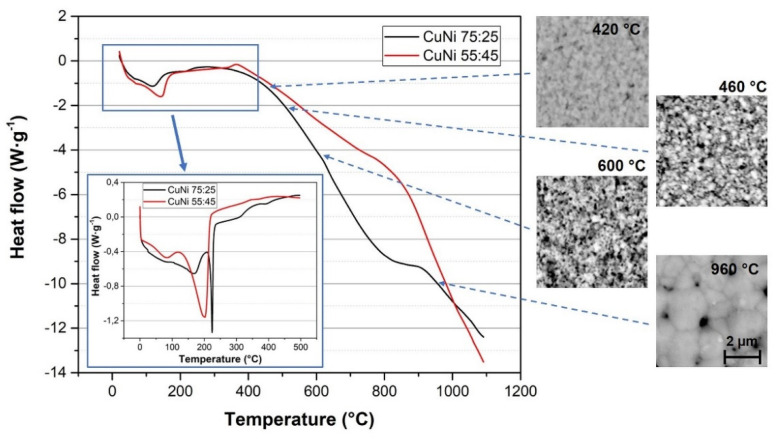
DSC analysis in N_2_ of prepared copper-nickel inks and results of test firings.

The DSC analyses of prepared inks were measured in the temperature range from 0 °C to 1100 °C by TA Instruments SDT Q600 and more accurately by TA Instruments DSC Q2000 in the limited temperature range up to 500 °C. The results of DSC analyses are shown in Figure 2. DSC analyses proved that inks contain three types of solvent that evaporate during firing. This evaporation represent endothermic peaks in the temperature range from 50 °C to 250 °C. At a temperature from 400 °C to 600 °C, the copper–nickel film does not sinter yet, which corresponds with results of test firings at temperatures of 420 °C and 600 °C (incoherent copper–nickel film, Figure 2). Both inks are sintered successfully and create a homogenous copper–nickel film at a temperature of around 960 °C (Figure 2). In the case of ink CuNi 75:25 prepared from copper and nickel nanoparticles, there is no visible endothermic peak at the melting point of copper at 1083 °C on the heat flow curve, which means that copper and nickel particles were well sintered and the copper–nickel alloy was formed. The copper–nickel alloy has a higher melting point than copper, 1160–1230 °C in the case of 75:25 copper–nickel alloy [21]. There is a visible difference between two resistive inks in terms of sintering. The ink composed of copper and nickel nanoparticles was better sintered at lower temperatures compared to ink composed of copper–nickel alloy nanoparticles. This is probably caused by the fact that copper nanoparticles begin to diffuse into the nickel nanoparticles at lower temperatures, and thus forming the copper–nickel alloy is more facile. In the case of the copper–nickel alloy, nanoparticles sintering is more difficult due to the higher melting point of the constantan alloy itself [21]. Nevertheless, using nanoparticles decreases the melting point compared to bulk material depending on the size of nanoparticles and the ratio of copper and nickel in alloy [22].

**Figure 3:**
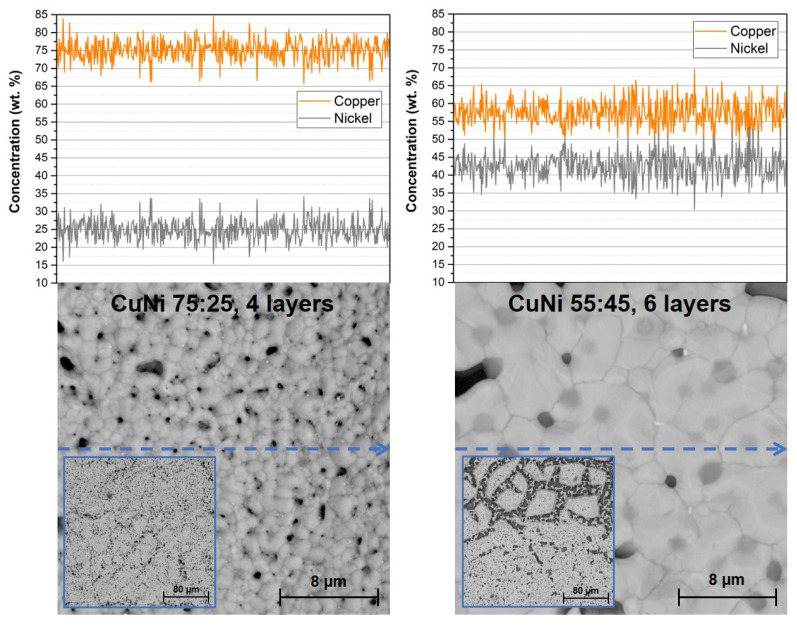
SEM images and EDS analyses of element distribution (line scan) in copper–nickel films.

The resistance and TCR values of produced specimens are summarized in Table 1. The comparable thickness of copper–nickel films could not be achieved with the same printing and firing process. In the case of ink CuNi 75:25, the deposition of four CuNi layers in one step was possible. The individual CuNi layers were dried directly during the deposition on the heated table with temperature 80 °C, which is a part of the Aerosol Jet device. The CuNi film was also dried at 130 °C for 10 min in the air after the deposition and fired in nitrogen at 960 °C with 10 min dwell time at the peak temperature. In the case of ink CuNi 55:45 prepared from constantan nanoparticles, the deposition of multiple layers was necessary and also multiple firings were needed due to the minimalization of crack formation. The printing and firing sequence was following: the printing of one CuNi layer, drying at 130 °C in air, firing at 960 °C in nitrogen, the printing of two CuNi layers, drying at 130 °C in air, firing at 960 °C in nitrogen, the printing of three CuNi layers, drying at 130 °C in air, firing at 960 °C in nitrogen—a total of six CuNi layers. Despite this sequential printing and firing process, cracks on the edges of the resistive film were formed (Figure 3).

The sheet resistance of specimens with CuNi 75:25 was ~0.1 Ω/square, and the sheet resistance of specimens with CuNi 55:45 was ~1 Ω/square. The resistance differences are caused by different copper–nickel ratios because the bulk copper–nickel 55:45 alloy has 1.5 times higher resistivity than the copper–nickel 75:25 alloy [23] and also by the formation of cracks on the edges of resistive film in the case of CuNi 55:45 ink (Figure 3). On the contrary, CuNi 55:45 has almost two times lower TCR (±100·10^−6^ K^−1^). These TCR values are satisfactory for printed resistors for intended applications in power electronic modules.

**Figure 4:**
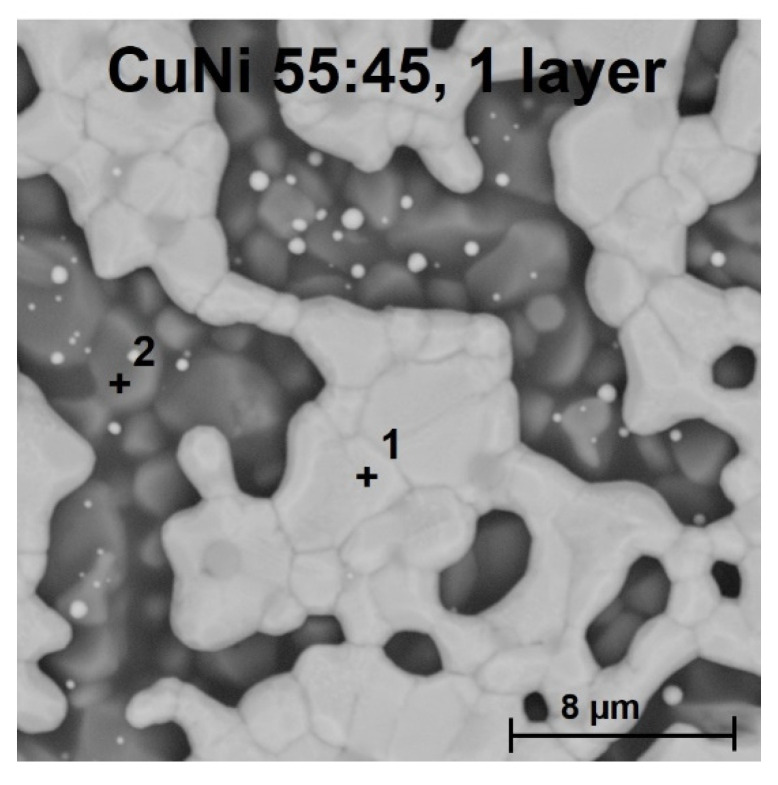
SEM image of the copper–nickel film—CuNi 55:45 ink, 1 layer, 1 firing—1: CuNi film, 2: Al_2_O_3_ substrate.

Fired copper–nickel films were observed by a Phenom ProX SEM (Waltham, MA, USA) microscope and copper–nickel ratios were verified by EDS analysis (line scan with a resolution of 512 points—Figure 3). Both types of inks have a homogenous composition of copper and nickel along the measured area. In the case of the CuNi 75:25 ink, even the distribution of copper and nickel in the final alloy occurred during firing. The CuNi 75:25 ink formed a fine-grained structure with grain size around 1 µm while CuNi 55:45 ink formed a better-sintered film having larger grains with size around 10 µm. The grain size of CuNi 55:45 ink is not affected by multiple firings; see the comparison of films with one layer/one firing and films with six layers/three firings—Figure 3 and Figure 4. The copper–nickel film thickness was ~2 µm for one layer/one firing and the continuous film was not formed (Figure 4).

## 4. Conclusions

It was verified using the IRP that the contact resistance of the produced resistor structures according to the nominal resistance value is significantly lower compared to the contact resistance of specimens of resistors with copper terminals formed using of conventional resistive pastes [8]. The number of contacts between copper–nickel resistive film and copper film does not affect the final TCR value. The TCR value is a crucial parameter of resistors; therefore, the CuNi 55:45 ink based on constantan nanoparticles is more appropriate for the production of resistors. Low sheet resistance ~1 Ω/square and low TCR ±100·10^−6^ K^−1^ values enable using these resistors as shunt resistors or current sensing resistors in power electronics modules. The nominal resistance value can be modified by copper–nickel film thickness and by the geometry of resistive film.

The compatibility with copper films is ensured thanks to the firing of the copper–nickel resistive film in a protective nitrogen atmosphere. The compatibility of copper–nickel and copper films permits the formation of integrated resistors directly on ceramics substrates of power electronics modules made by TPC technology. Copper–nickel and copper films can also be co-fired, which results in the reduction in production costs.

## 5. Patents

The invented method of producing a resistor for power applications, using Aerosol Jet printing technology to produce a resistor on a ceramic substrate, is protected by the European patent WO2021175347A1 Method of producing a resistor for power applications and national patent CZ308757B6 Resistor manufacturing method for power applications. This invention simplifies the production process by combining resistor film firing and the firing of electrically conductive patterns on a ceramic substrate in a single firing step at temperatures ranging from 650 °C to 960 °C in the same atmosphere.

## Figures and Tables

**Table 1:** Test specimens and results (average of 5 samples for each specimen).

Specimen		Number of CuNi Films	Number of Firings	Total Thickness of CuNi Film (µm)	Sheet Resistance (Ω/Square) *	TCR (10^−6^ K^−1^) **
CuNi 75:25	2 Cu-R contacts	4	1	~10	0.100	±186.8
	20 Cu-R contacts				0.143	±201.3
CuNi 55:45	2 Cu-R contacts	6 (1 + 2 + 3)	3	~10	1.017	±102.5
	20 Cu-R contacts				1.250	±100.9

* Statistical deviation, 5%. ** Statistical deviation, 10%.

## Data Availability

All data are described in the paper.

## References

[1] Rashid M.H. (2017). Power Electronics Handbook.

[2] Kaiser C.J. (1998). The Resistor Handbook.

[3] Seager C.W., Kokini K., Trumble K., Krane M.J.M. (2002). The Influence of CuAlO_2_ on the Strength of Eutectically Bonded Cu/Al_2_O_3_ Interfaces. Scr. Mater..

[4] Ning X.S., Lin Y., Xu W., Peng R., Zhou H., Chen K. (2003). Development of a Directly Bonded Aluminum/Alumina Power Electronic Substrate. Mater. Sci. Eng. B Solid-State Mater. Adv. Technol..

[5] Walker C.A., Hodges V.C. (2008). Comparing Metal-Ceramic Brazing Method. Weld. J..

[6] Van De Walle G. (1998). Integration of Passive Components: An Introduction. Philips J. Res..

[7] Jiang J.S., Liang J.E., Yi H.L., Chen S.H., Hua C.C. (2016). Performances of Screen-Printing Silver Thick Films: Rheology, Morphology, Mechanical and Electronic Properties. Mater. Chem. Phys..

[8] Hlina J., Reboun J., Johan J., Simonovsky M., Hamacek A. (2019). Reliability of Printed Power Resistor with Thick-Film Copper Terminals. Microelectron. Eng..

[9] Reboun J., Hlina J., Totzauer P., Hamacek A. (2018). Effect of Copper- and Silver-Based Films on Alumina Substrate Electrical Properties. Ceram. Int..

[10] Heraeus Electronics Lead Free Thick Copper Conductor System. https://www.heraeus.com/media/media/het/doc_het/products_and_solutions_het_documents/thick_film/data_sheets_th/Conductors_C7403_C7404A.pdf.

[11] Reboun J., Hromadka K., Hermansky V., Johan J. (2017). Properties of Power Electronic Substrates Based on Thick Printed Copper Technology. Microelectron. Eng..

[12] Rotman F., Navarro D., Mellul S. (1991). Optimised Nitrogen-Based Atmospheres for Copper Thick Film Manufacture: Part 1: Monitoring of Oxygen Doping in Nitrogen. Microelectron. Int. An Int. J..

[13] Hlina J., Reboun J., Hermansky V., Simonovsky M., Johan J., Hamacek A. (2019). Study of Co-Fired Multilayer Structures Based on Thick Printed Copper Technology. Mater. Lett..

[14] Hlina J., Reboun J., Hamacek A. (2020). Study of Copper Thick Film Metallization on Aluminum Nitride. Scr. Mater..

[15] Sergent J.E., Harper C.A. (1995). Hybrid Microelectronics Handbook.

[16] Kshirsagar A., Rane S., Mulik U., Amalnerkar D. (2007). Microstructure and Electrical Performance of Eco-Friendly Thick Film Resistor Compositions Fired at Different Firing Conditions. Mater. Chem. Phys..

[17] Davis J.R. (2001). Copper and Copper Alloys.

[18] Carreras A.C., Cangiano M.D.L.A., Ojeda M.W., Ruiz M.D.C. (2015). Characterization of Cu-Ni Nanostructured Alloys Obtained by a Chemical Route. Influence of the Complexing Agent Content in the Starting Solution. Mater. Charact..

[19] Cooper C., Hughes B. (2020). Aerosol Jet Printing of Electronics: An Enabling Technology for Wearable Devices. 2020 Pan Pacific Microelectron Symposium (Pan Pacific).

[20] Optomec Inc Aerosol Jet 300 Series Systems. https://www.optomec.com/wp-content/uploads/2014/04/AJ-300-Datasheet_Web.pdf.

[21] Féron D. (2007). Corrosion Behaviour and Protection of Copper and Aluminium Alloys in Seawater.

[22] Sopousek J., Vrestal J., Pinkas J., Broz P., Bursik J., Styskalik A., Skoda D., Zobac O., Lee J. (2014). Cu-Ni Nanoalloy Phase Diagram—Prediction and Experiment. Comput. Coupling Phase Diagr. Thermochem..

[23] Bakker D.J., Noat Y., Yanson A.I., van Ruitenbeek J.M. (2002). Effect of Disorder on the Conductance of a Cu Atomic Point Contact. Phys. Rev. B Condens. Matter Mater. Phys..

